# Microguidewire stiffness for microcatheter and aspiration catheter navigation in tortuous vessels

**DOI:** 10.1177/15910199251352883

**Published:** 2025-06-30

**Authors:** Kenichi Sakuta, Yoshiki Hanaoka, Mahsa Ghovvati, Amir Molaie, Taichiro Imahori, Keiko A Fukuda, Satoshi Tateshima, Naoki Kaneko

**Affiliations:** 1 Department of Neurology, David Geffen School of Medicine at UCLA, Los Angeles, CA, USA; 2 Department of Neurology, 26799 Jikei University School of Medicine, Tokyo, Japan; 3 Department of Radiological Sciences, 8783 David Geffen School of Medicine at UCLA, Los Angeles, CA, USA; 4 Department of Neurosurgery, 34808 Shinshu University School of Medicine, Matsumoto, Japan; 5 Department of Neurosurgery, Kitaharima Medical Center, Hyogo, Japan; 6 Center for Clinical Research and Innovation, Kobe City Medical Center General Hospital, Kobe, Japan

**Keywords:** Device navigation, microguidewire, microcatheter, aspiration catheter, tortuous anatomy

## Abstract

**Background:**

Effective catheter navigation and trackability are crucial in neuroendovascular procedures, particularly through tortuous vessels where catheter kickback can potentially delay treatment and worsen patient outcomes. While operational experience suggests that stiffer microguidewires enhance catheter navigation and trackability, this relationship has not been experimentally validated. This study assesses the impact of microguidewire stiffness on targeted catheter delivery using in-vitro vascular models with severe tortuosity.

**Methods:**

Two experiments were conducted using silicone models of the intracranial vasculature to evaluate microguidewires of similar composition but differing stiffness, as defined by greater resistance to bending with the Stryker Synchro Select Soft, Standard, and Support microguidewires. In Experiment 1, 0.021″ microcatheter navigation through an acute angle M2 branch of the middle cerebral artery was assessed. In Experiment 2, 0.071″ aspiration catheter navigation through a severely tortuous internal carotid artery model was tested. Maximum catheter pushing force and microguidewire kickback length were measured in both experiments.

**Results:**

Stiffer microguidewires required significantly lower pushing forces and exhibited reduced microwire kickback during both microcatheter and aspiration catheter advancement.

**Conclusions:**

Microguidewire stiffness significantly influences neuroendovascular catheter deliverability. Stiffer microguidewires provide greater system stability, particularly at the distal end, enhancing catheter navigation and advancement through tortuous anatomy.

## Introduction

Neuroendovascular therapy is a cornerstone treatment for cerebrovascular disorders, including acute ischemic stroke (AIS) and aneurysm embolization. With the introduction of stent retrievers and aspiration catheters, mechanical thrombectomy (MT) has become a widely performed procedure for large vessel occlusions (LVO) and is under continued randomized trial assessment of distal, medium vessel occlusions (DMVO).^1–9^ As rapid reperfusion improves outcomes, efficient delivery of microcatheters, stent retrievers, and aspiration catheters to target sites is critical.^10–14^

However, catheter navigation through tortuous anatomy, commonly seen in older patients with more cardiovascular risk factors, remains challenging. Acute angles can cause catheter kickback and buckling, delaying treatment or even preventing access.^15–17^ Failure to track a microcatheter or aspiration catheter past the ophthalmic segment of the internal carotid artery (ICA) has been linked to unsuccessful recanalization,^18^ while severe tortuosity is associated with procedural complications during MT for AIS.^19^ Hence, understanding effective techniques for catheter navigation through tortuosity in MT is necessary for enhancing treatment results.

Selecting the appropriate microguidewire in each case requires balancing properties such as torqueability, pushability, and stiffness. Operator experience suggests stiffer wires improve catheter trackability to its target site, but few studies have systematically tested this in realistic models. This study quantifies the impact of microguidewire stiffness on catheter navigation using in-vitro vascular models designed to mimic severe tortuosity.

## Methods

### In-vitro vascular model

Two silicone vascular models representing the ICA, anterior cerebral artery (ACA), and middle cerebral artery (MCA) were used to assess the impact of microguidewire stiffness on catheter navigation. The in-vitro experimental setup was prepared, as previously described.^20,21^ A pulsatile pump (Harvard Apparatus, MA, US) generated physiological flow within a closed-loop system filled with saline mixed with Tween 20 (polysorbate 20, a non-ionic surfactant added to reduce surface tension to simulate vascular conditions) at a concentration of 0.02% to simulate in vivo surface tension. A flow rate of 240 mL/min for the ICA, pulse rate of 60 beats per minute, and temperature of 37°C were maintained.

**Figure 1.**
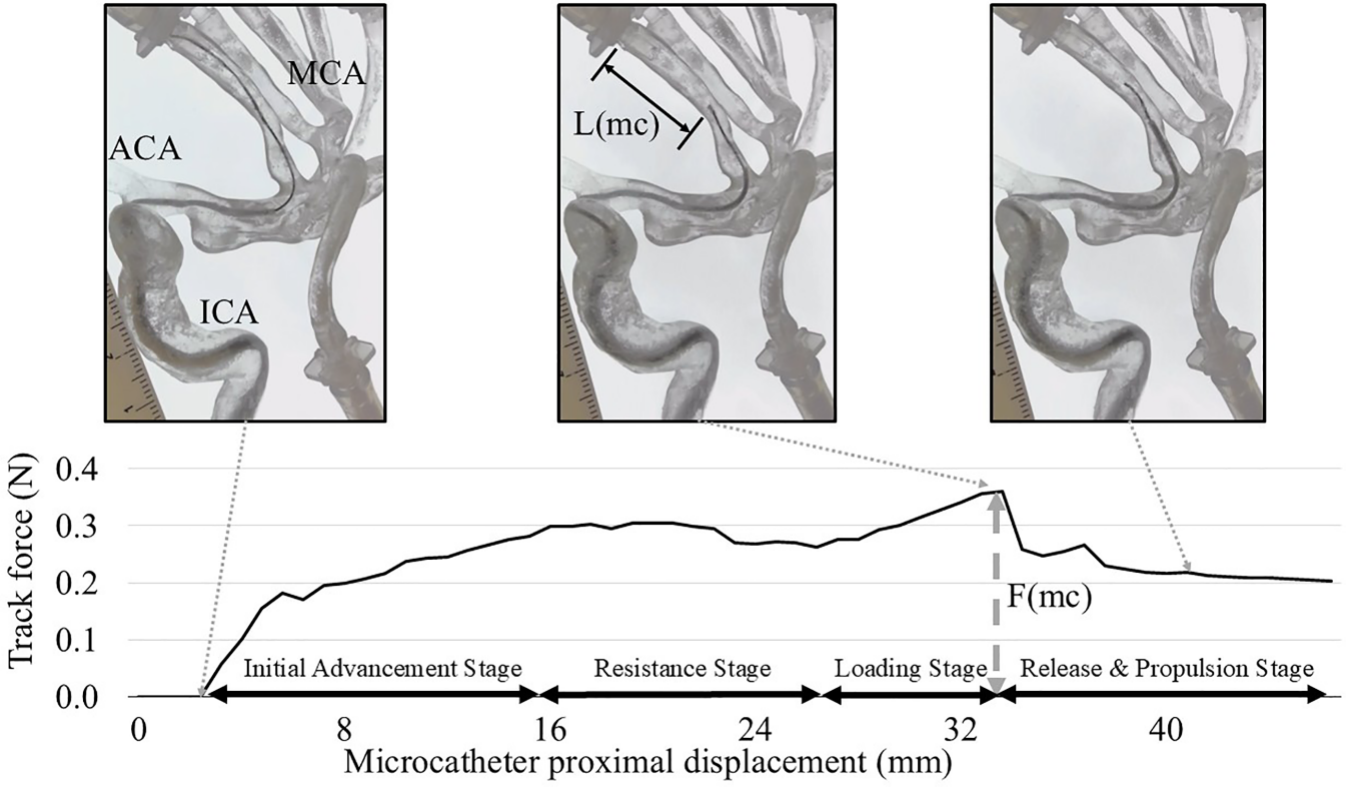
Microcatheter navigation in the middle cerebral artery (MCA) model with M2 branches. A microguidewire (Synchro Soft, Standard, or Support) with various stiffness was positioned in the prefrontal branch at an acute angle of 70°. The microcatheter was advanced at a rate of 4 mm/sec from the mid-M1 segment over the microguidewire toward the prefrontal branch. Stages of microcatheter advancement: (1) Initial Advancement Stage, as the microcatheter is advanced without resistance; (2) Resistance Stage, as the catheter tip remained stationary due to resistance despite proximal pushing force; (3) Loading Stage, wherein microguidewire kickback occurs while the pushing force gradually increased, and (4) Release and Propulsion Stage, at which point the peak force is reached, with the microcatheter advancing distally. The maximum microguidewire kickback length (L(mc)) in the M2 branch and the maximum pushing force (F(mc)) required to advance the microcatheter were measured.

**Figure 2.**
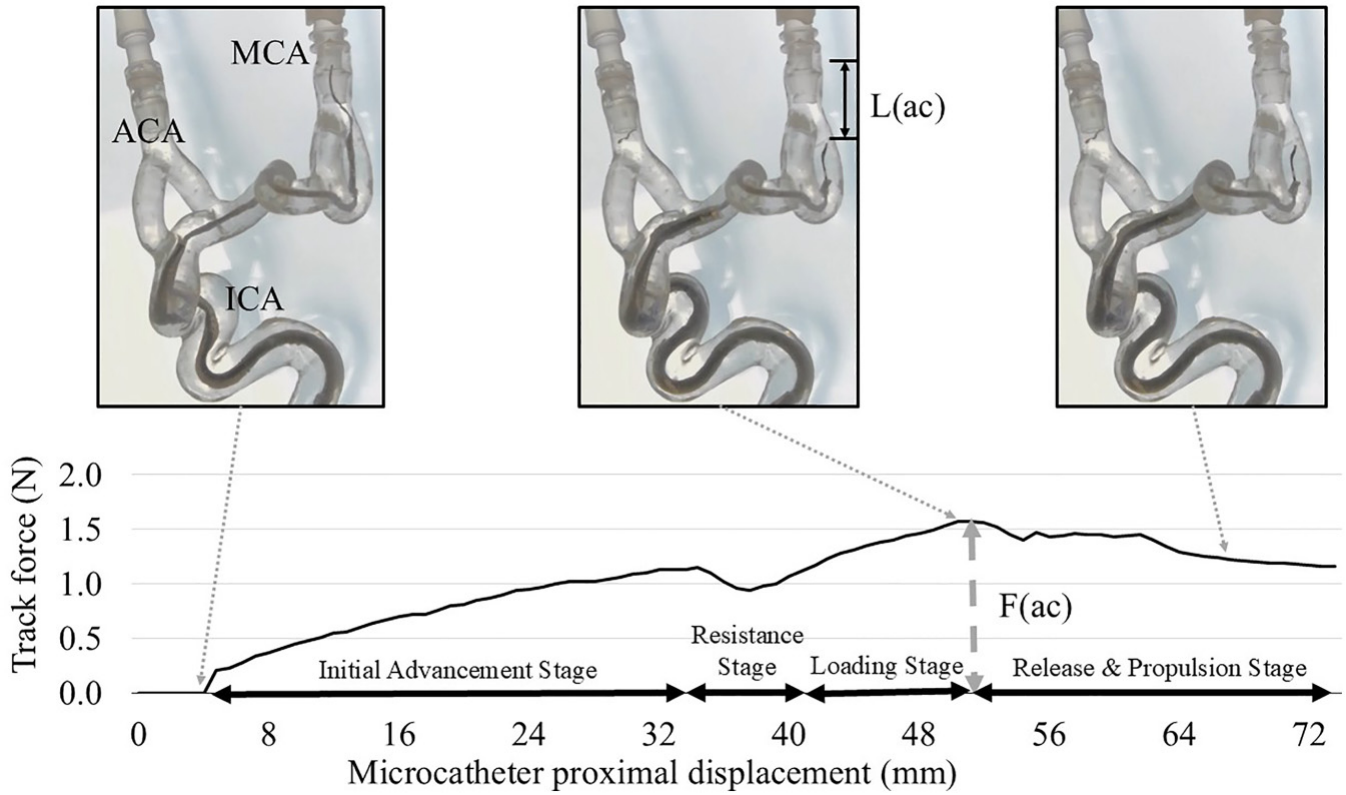
Aspiration catheter navigation in the internal carotid artery (ICA) model with severe tortuosity. The microcatheter and microguidewire were positioned in the inferior M2 branch, and the aspiration catheter was advanced at a rate of 4 mm/sec over the inner microcatheter from the ophthalmic segment of the ICA to the distal M1 segment. Stages of aspiration catheter advancement: (1) Initial Advancement Stage: the aspiration catheter is advanced to the ICA terminus without resistance; (2) Resistance Stage: the catheter tip remains stationary due to resistance at the acute bend over ICA terminus and M1 despite hand-side pushing force; (3) Loading Stage: the microguidewire and microcatheter experience kickback while the pushing force increases; and (4) Release and Propulsion Stage: the aspiration catheter advances distally once peak force is reached, accompanied by a decrease in force. The maximum microguidewire kickback length (L(ac)) in the M2 branch and the maximum pushing force (F(ac)) required to advance the aspiration catheter were measured.

The first model simulated an M2 branch of the MCA with a 70° acute angle (Figure 1). The second model simulated the ICA with severe tortuosity (Figure 2). Both models were designed using anatomical data and previously validated geometric parameters.^22,23^ The designation of “severe” tortuosity in the present ICA model is based on semi-automated calculations performed using an open-source package, Better Skeletonization, in MATLAB (MathWorks, Natick, Massachusetts, USA), as described previously.^24,25^ The pressure applied to the catheters was measured using a digital force gauge (DST-11A, IMADA, Japan).

### Microguidewires

Synchro Select 0.014″ microguidewires (Stryker Neurovascular, Fremont, CA), some of the most commonly used microguidewires in neuroendovascular procedures, were tested in this study. Synchro Select 0.014″ microguidewires are made with a stainless steel proximal core wire surrounded by a nitinol distal hypotube. Synchro Soft, Standard, and Support wires differ based on modified core designs which confer graduated stiffness based on their three-point bend forces along the distal 35 cm segment.^26^ Each are 0.014 in (0.36 mm) in width and 215 cm in length with the same distal 1 cm tip per the manufacturer. The distal tip of each microguidewire was kept in its straight shape configuration.

### Experiment 1: microcatheter navigation in the MCA model

The MCA model with M2 branches was used to assess the navigation of the microcatheter over the microguidewire (Figure 1). A coaxial system was prepared using an 8 Fr Shuttle guiding sheath (Cook Medical, Bloomington, IN) in the cervical ICA to provide excellent proximal support, a Zoom 71 aspiration catheter (Imperative Care Inc., Campbell, CA), and a Trevo Trak 21 microcatheter (Stryker Neurovascular, Fremont, CA). The microguidewire was advanced into the distal prefrontal M2 branch, while the microcatheter started in the proximal M1 segment. The aspiration catheter was stationed in the carotid siphon.

A digital force gauge connected to the microcatheter's proximal end measured the pushing force as an automated advancing device pushed the microcatheter at a constant rate of 4 mm/sec. Force was recorded until the microcatheter tip reached the mid-M2 branch. The positions of the microguidewire, microcatheter, and aspiration catheter were standardized to ensure consistency during each experiment. The maximum kickback length of the microguidewire (Max L(microcatheter (mc)), as well as the maximum pushing force (Max F(mc)), was measured from video recordings.

### Experiment 2: aspiration catheter navigation in the ICA model

The same coaxial setup (8 Fr Shuttle, Zoom 71, and a Trevo Trak 21) was used in an ICA model with severe tortuosity to evaluate the navigation of the aspiration catheter (Figure 2). Either a Synchro Select 0.014″ Soft, Standard, or Support microguidewire was advanced into the distal M2 segment, while the microcatheter was placed in the mid-M2 and the aspiration catheter was placed in the carotid siphon. A digital force gauge connected to the proximal end of the aspiration catheter measured the pushing force applied during advancement (4 mm/sec). The force was measured until the tip of the aspiration catheter reached the M2 bifurcation. The maximum kickback length of the microguidewire and aspiration catheter (Max L(ac)), as well as the maximum pushing force applied to the aspiration catheter (Max F(ac)), was measured from video recordings.

### Statistical analyses

Each test was repeated seven times to maximize accuracy, reliability, and power of our data while working under the constraints of device availability. Results are reported as mean ± standard deviation (SD). One-way analysis of variance (ANOVA) was used to compare the maximum pushing forces and kickback lengths across the different microguidewires. Tukey's honestly significant difference (HSD) test was performed for post hoc comparisons. A p-value of less than 0.05 was considered statistically significant. All statistical analyses were conducted using SPSS statistical software (version 23 for Windows; SPSS Inc., Chicago, IL, USA).

## Results

### Experiment 1: Microcatheter navigation in the MCA model

Consistent patterns of device behavior were observed regardless of microguideiwre stiffness as illustrated in the following stages for both experiments: (1) *Initial Advancement Stage*, the microcatheter advanced up to the bifurcation point, which is the origin of the M2 prefrontal branch (asterisk in Figure 1(a)); (2) *Resistance Stage*, although the proximal end of the microcatheter (hand side) was being advanced by the pushing machine, the tip remained stationary due to resistance at the acute bend; (3) *Loading Stage*, the force required to advance the microcatheter tip over the acute angle gradually increased as the microguidewire was pushed back (Figure 1(b)); and (4) *Release and Propulsion Stage*, after the necessary force (peak force) was reached, both the microcatheter and microguidewire rapidly advanced into the prefrontal M2 branch, accompanied by a decrease in force (Figure 1(c)).

**Figure 3.**
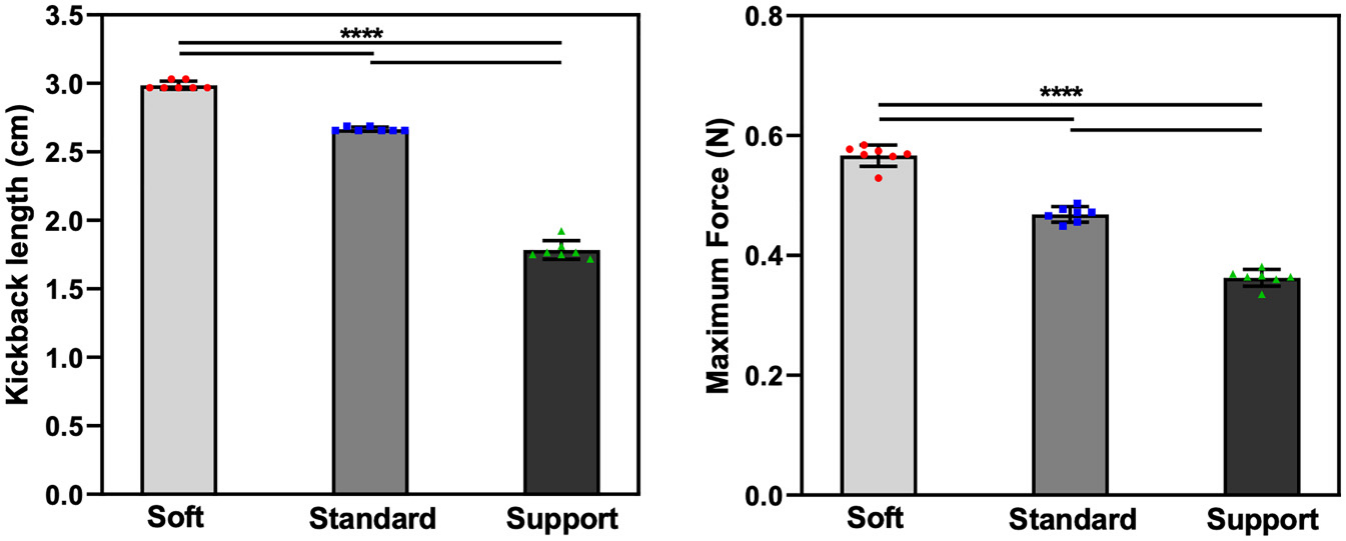
Impact of microguidewire stiffness on microcatheter navigation in the M2 branch with an acute angle. (Left) Kickback length (L(mc)) of the microguidewire in the M2 branch for Soft, Standard, and Support microguidewires during the microcatheter advancement. (Right) Maximum force (F(mc)) required to advance the microcatheter over the microguidewire. Both the kickback length and the maximum force significantly decreased as microguidewire stiffness increased (*P* < 0.001). Data are presented as mean ± SD, and statistical significance is indicated by **** (*P* < 0.0001).

The peak force F(mc) applied to the proximal end of the microcatheter and the length of microguidewire kickback, L(mc), across different levels of microguidewire stiffness (Figure 3) was measured. The mean hand-side pushing forces required to advance the microcatheter were 0.567 N (Synchro Soft), 0.468 N (Synchro Standard), and 0.363 N (Synchro Support) (*P* < 0.001). Similarly, the measured microguidewire kickback lengths were 2.987, 2.665, and 1.783 cm, respectively (*P* < 0.001). These findings indicate that stiffer microguidewires provide superior proximal support, significantly reducing both the pushing force required for microcatheter advancement and the degree of wire kickback.

### Experiment 2: Navigation of the aspiration catheter in the ICA

The following consistent behaviors were observed within a highly tortuous ICA model (Figure 2): (1) *Initial Advancement Stage*, the aspiration catheter was advanced to the ICA terminus without resistance; (2) *Resistance Stage*, although the proximal end of the aspiration catheter (hand side) was being advanced by the pushing machine, the catheter tip remained stationary due to resistance at the acute bend over ICA terminus and M1; (3) *Loading Stage*, the microguidewire and microcatheter experienced a kickback while the pushing force was gradually increasing (Figure 2(a) and (b)); and (4) *Release and Propulsion Stage*, after the necessary force (peak force) was reached to advance the aspiration catheter (asterisk in Figure 2(b)), the aspiration catheter advanced distally, accompanied by a decrease in force (Figure 2(c)).

**Figure 4.**
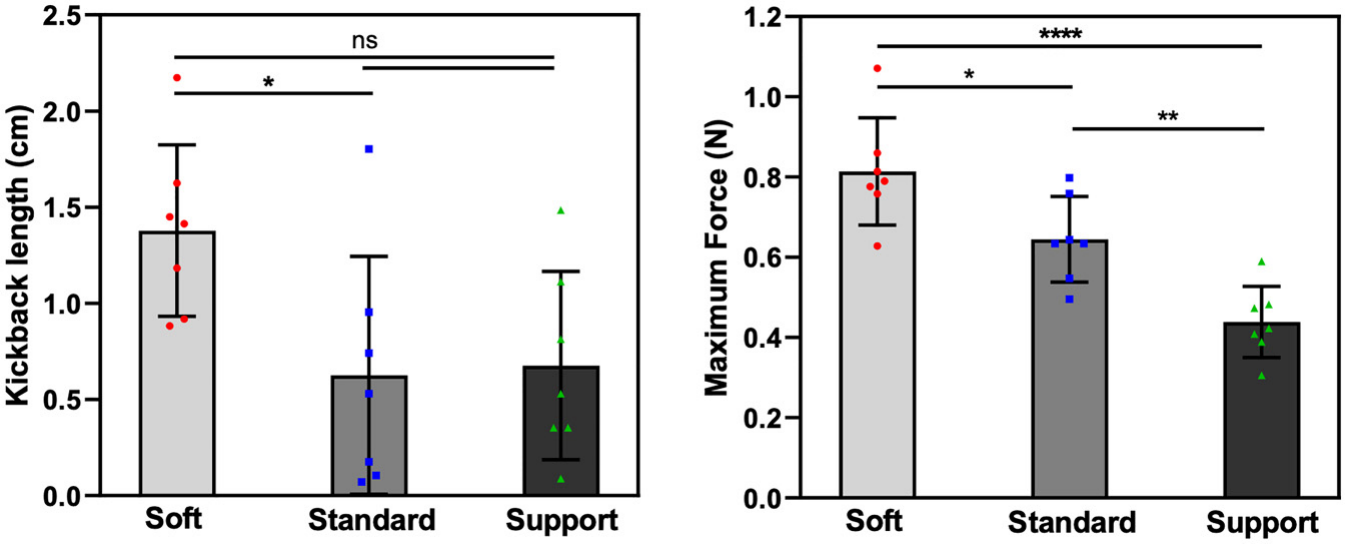
Impact of microguidewire stiffness on aspiration catheter navigation in a tortuous ICA model. (Left) Kickback length (L(ac)) of the microguidewire in the M2 branch for Soft, Standard, and Support microguidewires. (Right) Maximum force (F(ac)) required to advance the aspiration catheter. Both the kickback length and the maximum force significantly decreased as microguidewire stiffness increased (*P* < 0.05). Data are presented as mean ± SD, with statistical significance indicated by **** (*P* < 0.0001), ** (*P* < 0.01), * (*P* < 0.05), and ns (not significant).

The peak force F(ac) applied to the proximal end of the aspiration catheter and the kickback length L(ac) at the point of resumed forward movement in the microguidewires was measured (Figure 4). The mean hand-side pushing forces required to advance the aspiration catheter were 0.813 N (Synchro Soft), 0.645 N (Synchro Standard), and 0.439 N (Synchro Support) (*P* < 0.001). The kickback length of the catheters were 1.378, 0.626, and 0.676 cm, respectively (*P* = 0.026). Similarly, microguidewire stiffness significantly improved aspiration catheter trackability and microguidewire kickback length.

## Discussion

This study demonstrated that microguidewire stiffness significantly affects the ease of delivery of commonly used microcatheters and aspiration catheters in tortuous neurovascular anatomy. As microguidewire stiffness increased, both the force needed to advance the catheters and wire kickback lengths decreased, highlighting improved catheter support and system stability. Conversely, device herniation and slack were observed with softer microguidewires, with greater force needed to advance the catheters and a larger wire kickback. These findings validate the long-standing assumption among neurointerventionalists that stiffer microguidewires improve enhance device trackability. These results have not been previously demonstrated experimentally.

Effective catheter navigation is an important determinant of procedural success in neuroendovascular interventions. This requires careful and precise selection of a microguidewire tailored to the anatomical context, balancing key characteristics such as trackability, pushability, torqueability, flexibility, and clot crossability. However, these attributes oftentimes work against each other. For example, using the same wire optimized for tracking up stiff support systems can be potentially harmful when performing delicate catheterizations.^27^ Therefore, understanding each mechanical property in isolation helps interventionalists optimize device selection based on the key procedural needs at hand.

The mechanical stiffness of a microguidewire is largely attributed to the flexural modulus of its core metal composition (e.g. stainless steel, nitinol),^28^ as well as its outer diameter.^29–31^ Methodology such as the “three-point bend test” has been devised to better elucidate the flexural rigidity profiles of neuroendovascular devices, including guidewires, intermediate catheters, and guiding sheaths though little is published regarding the comparative stiffness of microwires.^26,31^ Our experiments showed that a stiffer microguidewire enhanced support, which reduced the tendency of the guidewire to buckle at acute angles and, in turn, minimized the slack in the catheter system.

While softer microguidewires may appear safer for traversing complex anatomy, their greater risk of herniation and slack in the system require greater force for microcatheter tracking, thereby reducing precision and increasing the risk of unintended herniation into neighboring vessels. Schulze-Zachau et al. reported that approximately half of all perforations during MT were related to device access.^33^ Sudden forward movements of a microguidewire and microcatheter, as observed in the Release and Propulsion Stages of the present study, can increase the risk of vascular perforation. In addition, frequent device kickback and herniation may decrease procedural safety and increase the procedural time.

Additionally, Li et al. previously reported that distal access catheters require 30% more force when tracking without microcatheter support.^32^ Our findings extend this principle to the microguidewire itself, showing that wire stiffness similarly plays an important role in enhancing support during catheter advancement, allowing for faster and safer device navigation in neurointerventional procedures. Notably, guidewires with a stiffer distal portion may also increase the risk of vessel injury including dissection, perforation, or vasospasm, particularly in delicate or distal vasculature. Therefore, extra care must be taken when navigating in distal vasculature.^34,35^

Safe navigation of devices is also important in aneurysm treatment. Ryu et al. previously noted that all cases of vascular perforation during aneurysm coil embolization were related to guidewire navigation.^36^ Kawabata et al. also found that 20% of intraprocedural ruptures during unruptured cerebral aneurysm treatment occurred during device access.^37^ Although Synchro Select wires come in different stiffness profiles, their distal-most tips have similar softness, as dictated by their distal three point bend force.^26^ While softer wires may seem safer for aneurysm access, a soft microwire may lead to system instability such as unintended forward device motion around the aneurysm. For cases involving sidewall aneurysms with acute angles from the parent artery or for navigating relatively stiff microcatheters for intrasaccular devices, choosing a supportive wire such as Synchro Standard or Support may help reduce procedural complications by providing better stability during navigation to aneurysm.

In the MCA model, both the maximum microguidewire kickback length (L(mc)) in the M2 branch and the maximum pushing force (F(mc)) required to advance the microcatheter forward sequentially increased from the Synchro Support wire, to the Synchro Standard wire, followed by the Synchro Soft wire. However, in the ICA model, though the F(ac) required to advance the Zoom 71 successively increased in the same manner, the kickback length L(ac) did not follow the same pattern between the Synchro Standard and Synchro Support wires, with larger confidence intervals observed (Figure 4). The reason behind this inconsistency is unclear, though may be related to slight micromillimeter changes in initial AC position near the ophthalmic segment at the start of each trial, or simply small sample size. Future studies should consider either more proximal or distal positioning of the AC, with larger number of repetitions per experiment.

Recently, there has been a shift toward systems that obviate the need for microcatheter use particularly in MT for LVO. In particular, 0.035″ intracranial “macroguidewires” are being used in place of 0.014″ microwires^38,39^ and “delivery catheters” in place of microcatheters.^40,41^ It may be reasonable to hypothesize that the physical properties governing trackability over macroguidewires using delivery systems parallel those tested in the present study using the traditional coaxial setup with microguidewires and microcatheters. However, our findings should be further evaluated and validated using these newer catheter delivery systems in the future.

### Limitations

This study has several limitations. First, testing was performed using in-vitro silicone vascular models. While these are anatomically realistic, they may not replicate the mechanical properties of human arteries, such as elasticity and compliance. Second, only a limited selection of microguidewires was tested, which may not reflect the full range of devices used in clinical practice. Future studies are necessary to validate and generalize these findings to clinical practice.

## Conclusion

Microguidewire stiffness significantly influences catheter deliverability in tortuous neurovascular anatomy. Stiffer microguidewires provide superior system stability, reducing the force required for catheter advancement and decreasing microwire kickback. These benefits facilitate more efficient and controlled navigation, ultimately supporting safer and faster access to target sites in neuroendovascular procedures. Softer microguidewires required greater force and may cause kickbacks, which can increase procedural time. Optimal microguidewire selection, tailored to the specific anatomical and procedural context, is essential for enhancing procedural success and minimizing complications associated with tortuous vascular anatomies.

## Abbreviations

ACA: anterior cerebral artery
AIS: acute ischemic stroke
DMVO: distal medium vessel occlusions
ICA: internal carotid artery
LVO: large vessel occlusion
MCA: middle cerebral artery
MT: mechanical thrombectomy

## References

[1] Goyal M Menon BK van Zwam WH , et al. Endovascular thrombectomy after large-vessel ischaemic stroke: a meta-analysis of individual patient data from five randomised trials. Lancet (London, England) 2016; 387: 1723–1731. 26898852 10.1016/S0140-6736(16)00163-X

[2] Nogueira RG Jadhav AP Haussen DC , et al. Thrombectomy 6 to 24 hours after stroke with a mismatch between deficit and infarct. N Engl J Med 2018; 378: 11–21. 29129157 10.1056/NEJMoa1706442

[3] Jovin TG Chamorro A Cobo E , et al. Thrombectomy within 8 hours after symptom onset in ischemic stroke. N Engl J Med 2015; 372: 2296–2306. 25882510 10.1056/NEJMoa1503780

[4] Campbell BC Mitchell PJ Kleinig TJ , et al. Endovascular therapy for ischemic stroke with perfusion-imaging selection. N Engl J Med 2015; 372: 1009–1018. 25671797 10.1056/NEJMoa1414792

[5] Saver JL Goyal M Bonafe A , et al. Stent-retriever thrombectomy after intravenous t-PA vs. t-PA alone in stroke. N Engl J Med 2015; 372: 2285–2295. 25882376 10.1056/NEJMoa1415061

[6] Berkhemer OA Fransen PS Beumer D , et al. A randomized trial of intraarterial treatment for acute ischemic stroke. N Engl J Med 2015; 372: 11–20. 25517348 10.1056/NEJMoa1411587

[7] Rai AT Link PS Domico JR . Updated estimates of large and medium vessel strokes, mechanical thrombectomy trends, and future projections indicate a relative flattening of the growth curve but highlight opportunities for expanding endovascular stroke care. J Neurointerv Surg 2023; 15: e349–e355. 10.1136/jnis-2022-019777 PMC10803998 36564202

[8] Meyer L Stracke P Wallocha M , et al. Aspiration versus stent retriever thrombectomy for distal, Medium vessel occlusion stroke in the posterior circulation: a subanalysis of the TOPMOST study. Stroke 2022; 53: 2449–2457. 35443785 10.1161/STROKEAHA.121.037792

[9] Rodriguez-Calienes A Vivanco-Suarez J Sequeiros JM , et al. Mechanical thrombectomy for the treatment of primary and secondary distal medium-vessel occlusion stroke: systematic review and meta-analysis. J Neurointerv Surg 2023; 15: e460–e467. 10.1136/jnis-2022-019975 36797050

[10] Mulder M Jansen IGH Goldhoorn RB , et al. Time to endovascular treatment and outcome in acute ischemic stroke: mR CLEAN registry results. Circulation 2018; 138: 232–240. 29581124 10.1161/CIRCULATIONAHA.117.032600

[11] Bourcier R Goyal M Liebeskind DS , et al. Association of time from stroke onset to groin puncture with quality of reperfusion after mechanical thrombectomy: a meta-analysis of individual patient data from 7 randomized clinical trials. JAMA Neurol 2019; 76: 405–411. 30667465 10.1001/jamaneurol.2018.4510 PMC6459219

[12] Jahan R Saver JL Schwamm LH , et al. Association between time to treatment with endovascular reperfusion therapy and outcomes in patients with acute ischemic stroke treated in clinical practice. Jama 2019; 322: 252–263. 31310296 10.1001/jama.2019.8286 PMC6635908

[13] Mazighi M Chaudhry SA Ribo M , et al. Impact of onset-to-reperfusion time on stroke mortality: a collaborative pooled analysis. Circulation 2013; 127: 1980–1985. 23671178 10.1161/CIRCULATIONAHA.112.000311

[14] Ribo M Molina CA Cobo E , et al. Association between time to reperfusion and outcome is primarily driven by the time from imaging to reperfusion. Stroke 2016; 47: 999–1004. 26956258 10.1161/STROKEAHA.115.011721

[15] Koge J Tanaka K Yoshimoto T , et al. Internal carotid artery tortuosity: impact on mechanical thrombectomy. Stroke 2022; 53: 2458–2467. 35400203 10.1161/STROKEAHA.121.037904 PMC9311296

[16] Mokin M Waqas M Chin F , et al. Semi-automated measurement of vascular tortuosity and its implications for mechanical thrombectomy performance. Neuroradiology 2021; 63: 381–389. 32816090 10.1007/s00234-020-02525-6 PMC7880861

[17] Alverne F Lima FO Rocha FA , et al. Unfavorable vascular anatomy during endovascular treatment of stroke: challenges and bailout strategies. J Stroke 2020; 22: 185–202. 32635684 10.5853/jos.2020.00227 PMC7341011

[18] Singh J Wolfe SQ Janjua RM , et al. Anchor technique: use of stent retrievers as an anchor to advance thrombectomy catheters in internal carotid artery occlusions. Intervent Neuroradiol: J Perither Neuroradiol Surg Proc Relat Neurosci 2015; 21: 707–709. 10.1177/1591019915609170 PMC4757366 26494404

[19] Saber H Colby GP Mueller-Kronast N , et al. Arterial tortuosity is a potent determinant of safety in endovascular therapy for acute ischemic stroke. Stroke: Vascular Intervent Neurol 2024; 4: e001178. 10.1161/SVIN.123.001178 PMC12778508 41584471

[20] Kaneko N Mashiko T Ohnishi T , et al. Manufacture of patient-specific vascular replicas for endovascular simulation using fast, low-cost method. Sci Rep 2016; 6: 39168. 27976687 10.1038/srep39168 PMC5156941

[21] Kaneko N Takayanagi A Saber H , et al. A novel intracranial exchange guidewire improves the navigation of various endovascular devices: an in vitro study of challenging situations. Interv Neuroradiol 2022; 28: 588–594. 34787015 10.1177/15910199211057332 PMC9511615

[22] Griessenauer CJ Yalcin B Matusz P , et al. Analysis of the tortuosity of the internal carotid artery in the cavernous sinus. Childs Nerv Syst 2015; 31: 941–944. 25749877 10.1007/s00381-015-2674-x

[23] Yamamoto S Yamagami H Todo K , et al. Correlation of middle cerebral artery tortuosity with successful recanalization using the merci retrieval system with or without adjunctive treatments. Neurol Med Chir 2014; 54: 113–119. 10.2176/nmc.oa2012-0348 PMC4508709 24162242

[24] Kaneko N Komuro Y Yokota H , et al. Stent retrievers with segmented design improve the efficacy of thrombectomy in tortuous vessels. J Neurointerv Surg 2019; 11: 119–122. 30045949 10.1136/neurintsurg-2018-014061

[25] Kim BJ Yang E Kim N-Y , et al. Vascular tortuosity may be associated with cervical artery dissection. Stroke 2016; 47: 2548–2552. 27531344 10.1161/STROKEAHA.116.013736

[26] Neurovascular S . Synchro SELECT. Accessed 2/10/2025, 2025. https://www.stryker.com/us/en/neurovascular/products/synchro-select-guidewires.html .

[27] Molaie AM Sakuta K Hanaoka Y , et al. Abstract WP195: the impact of tortuosity on microwire torquability. Stroke 2024; 55: AWP195–AWP195.

[28] Harrison GJ How TV Vallabhaneni SR , et al. Guidewire stiffness: what's in a name? J Endovasc Ther 2011; 18: 797–801. 22149229 10.1583/11-3592.1

[29] Schröder J . The mechanical properties of guidewires. Part I: stiffness and torsional strength. Cardiovasc Intervent Radiol 1993; 16: 43–46. 8435835 10.1007/BF02603036

[30] Schroeder J . Peripheral vascular interventions : an illustrated manual . Thieme 2013.

[31] Qiu MY Suskin CB Becerra-Garcia JJ , et al. Quantification of the flexural rigidity of endovascular surgical devices using three-point bending tests. Res Sq 2023.

[32] Li J Tomasello A Requena M , et al. Trackability of distal access catheters: an in vitro quantitative evaluation of navigation strategies. J Neurointerv Surg 2023; 15: 496–501. 35450927 10.1136/neurintsurg-2022-018889

[33] Schulze-Zachau V Brehm A Ntoulias N , et al. Incidence and outcome of perforations during medium vessel occlusion compared with large vessel occlusion thrombectomy. J Neurointerv Surg 2024; 16: 775–780. 37524518 10.1136/jnis-2023-020531

[34] Pilgram-Pastor SM Piechowiak EI Dobrocky T , et al. Stroke thrombectomy complication management. J Neurointerv Surg 2021; 13: 912–917. 34158401 10.1136/neurintsurg-2021-017349 PMC8458081

[35] Goeggel Simonetti B Hulliger J Mathier E , et al. Iatrogenic vessel dissection in endovascular treatment of acute ischemic stroke. Clin Neuroradiol. 2019; 29: 143–151. 29098320 10.1007/s00062-017-0639-z PMC6394531

[36] Ryu CW Lee CY Koh JS , et al. Vascular perforation during coil embolization of an intracranial aneurysm: the incidence, mechanism, and clinical outcome. Neurointervention. 2011; 6: 17–22. 22125743 10.5469/neuroint.2011.6.1.17 PMC3214804

[37] Kawabata S Imamura H Adachi H , et al. Risk factors for and outcomes of intraprocedural rupture during endovascular treatment of unruptured intracranial aneurysms. J Neurointervent Surg. 2018; 10: 362–366. 10.1136/neurintsurg-2017-013156 28710085

[38] Tonetti D Koneru M Brinjikji W , et al. LB-011 Aristotle colossus guidewire in acute anterior circulation ischemic stroke thrombectomy: a multicenter experience. J Neurointerv Surg 2024; 16: A274–A275.

[39] Limaye K Al Kasab S Dolia J , et al. Macrowire for intracranial thrombectomy: an early experience of a new device and technique for anterior circulation large vessel occlusion stroke. Intervent Neuroradiol: J Peritherap Neuroradiol Surg Proc Rel Neurosci 2024: 15910199241308328. 10.1177/15910199241308328 PMC11659961 39692535

[40] Tonetti DA Bhattacharyya M Koneru M , et al. Novel tenzing 7 delivery catheter for thrombectomy in acute stroke: a clinical multicenter experience. Stroke: Vasc Intervent Neurol 2023; 3: e000940. 10.1177/15910199231216765 PMC13305372 38018024

[41] Dhar V Barrera P Akkipeddi S , et al. E-118 A single-center study of the RED 72 reperfusion catheter with SENDit technology in proximal large vessel occlusions. J Neurointerv Surg 2024; 16: A156–A156. 10.1177/15910199251358597 PMC12279763 40686308

